# Towards a dynamic list of Amazonian tree species

**DOI:** 10.1038/s41598-019-40101-y

**Published:** 2019-03-05

**Authors:** Hans ter Steege, Sylvia Mota de Oliveira, Nigel C. A. Pitman, Daniel Sabatier, Alexandre Antonelli, Juan E. Guevara Andino, Gerardo A. Aymard, Rafael P. Salomão

**Affiliations:** 1 Naturalis Biodiversity Center, Vondellaan 55, Postbus 9517, 2300 RA Leiden, The Netherlands; 2 Coordenação de Botânica, Museu Paraense Emílio Goeldi, Av. Magalhães Barata 376, C.P. 399, Belém, PA 66040–170 Brazil; 3 Systems Ecology, Free University Amsterdam, Amsterdam, The Netherlands; 4 Science and Education, The Field Museum, 1400S. Lake Shore Drive, Chicago, IL 60605–2496 USA; 5 AMAP, IRD, CIRAD, CNRS, Université de Montpellier, INRA, Boulevard de la Lironde, TA A-51/PS2, F-34398 Montpellier Cedex, France; 6 Department of Biological and Environmental Sciences, University of Gothenburg, Box 461, SE-405 30, Göteborg, Sweden; 7 Gothenburg Botanical Garden, Carl Skottsbergsgata 22A, SE-413 19 Göteborg, Sweden; 8 Gothenburg Global Biodiversity Centre, Box 461, SE-405 30 Göteborg, Sweden; 9 Department of Organismic and Evolutionary Biology, Harvard University, 26 Oxford St., Cambridge, MA 02138 USA; 10 Yachay Tech, School of Biological Sciences, Hacienda San José s/n, San Miguel de Urcuquí, Ibarra, Ecuador; 11 Grupo de Investigación en Biodiversidad, Medio Ambiente y Salud-BIOMAS- Universidad de las Américas, Campus Queri, Quito, Ecuador; 12 UNELLEZ-Guanare, Programa de Ciencias del Agro y el Mar, Herbario Universitario (PORT), Mesa de Cavacas, Estado Portuguesa, 3350 Venezuela; 13 Present Address: Compensation International Progress S.A., P.O. Box 260161 Bogotá, D. C. Colombia; 14 Programa Professor Visitante Nacional Sênior na Amazônia – CAPES, Universidade Federal Rural da Amazônia, Av. Perimetral, s/n, Belém, PA Brazil

## Abstract

To provide an empirical foundation for estimates of the Amazonian tree diversity, we recently published a checklist of 11,675 tree species recorded to date in the region (ter Steege H, *et al*. (2016) The discovery of the Amazonian tree flora with an updated checklist of all known tree taxa. Scientific Reports 6:29549). From this total of plant records compiled from public databases and literature, widely used in studies on the Amazonian plant diversity, only 6,727 tree species belong to the first taxonomically-vetted checklist published for the region (Cardoso D, *et al*. (2017) Amazon plant diversity revealed by a taxonomically verified species list. PNAS 114:10695-10700). The striking difference in these two numbers spurred us to evaluate both lists, in order to release an improved Amazonian tree list; to discuss species inclusion criteria; and to highlight the ecological importance of verifying the occurrence of “non-Amazonian” trees in the region through the localization and identification of specimens. A number of species in the 2016 checklist that are not trees, non-native, synonyms, or misspellings were removed and corresponded to about 23% of the names. Species not included in the taxonomically-vetted checklist but verified by taxonomists to occur in Amazonia as trees were retained. Further, the inclusion of recently recorded/new species (after 2016), and recent taxonomic changes added up to an updated checklist including 10,071 species recorded for the Amazon region and shows the dynamic nature of establishing an authoritative checklist of Amazonian tree species. Completing and improving this list is a long-term, high-value commitment that will require a collaborative approach involving ecologists, taxonomists, and practitioners.

## Introduction

Regional species lists are testable scientific hypotheses that are increasingly used in ecological and biodiversity research. Once primarily constructed and used by taxonomists, these lists (and hypotheses) became increasingly important in macroecology^1^, in studies of alpha and beta diversity patterns and conservation^2,3^, on estimations of species richness of undersampled areas^4,5^, and on community assembly research that includes the delimitation of a metacommunity^6^. The recent developments towards e-taxonomy allowed species records to be compiled from online databases using various bioinformatic resources e.g.^7–9^, however, not without risk of inaccurate data inclusion/exclusion.

Listing the flora of a given area involves area delimitation, which can be defined by political, geographical, or biome borders; as well as the choice of a focus group, which can be taxonomic or based on, for instance, plant habit^10^. Species inclusion criteria, therefore, must be clearly stated, in line with the goal of such an exercise. For instance, the inclusion of invasive or ornamentals may turn out desirable from ecological and regulatory points of view^11,12^, but not relevant in taxonomic monographs; and species records originally from an ecotone will be part of a geographical delimited list – potentially relevant for ecological studies – but might be rejected under landscape based delimitations.

In 2016 we published the first list of tree species recorded in Amazonia^5^, together with an historical overview of collections in the region from the 18^th^ century through the present, and a discussion of five caveats surrounding such an extensive list. Our intent was to encourage collaboration and debate between ecologists with a broad knowledge of the Amazonian tree flora and taxonomists with expertise in specific groups, with the ultimate goal being a curated and regularly updated list of Amazonian tree taxa. Subsequently, Cardoso *et al*.^4^ published the first taxonomically-vetted checklist of all Amazonian seed plant species, and argued that our work represented a severe overestimate of Amazonian tree diversity. Here we focus on the stark difference in tree species numbers reported by the two lists: 11,675^5^ vs. 6,727^4^ and set apart differences caused by errors from those that resulted from different species inclusion criteria or taxonomic opinion, which are further discussed.

In this article, we keep up the compromise of a long term effort towards a dynamic Amazonian tree species list, by (1) summarizing the corrections we have implemented thanks to Cardoso *et al*.’s article; (2) discussing the scientific grounds to retain a large number of species; (3) moving towards a hypothesis based list, in which confidence levels for the inclusion of species are clearly defined, improving its quality and broadening its usability.

## Results and Discussion

The reassessment of our list of Amazonian tree species^5^ resulted in an updated list of 10,071 species (SI Appendix 1, Checklist of Amazonian Tree Species), and a large number of taxonomic changes (SI Appendix 2, synonyms changed). A total of 864 spelling variants and synonyms were removed. From the 10,071 species names, 8049 attend to confidence level 1 (taxonomically vetted), 590 attend to level 2 (reported for Amazon in Reflora)^13^, and 1432 to level 3 (reported in Amazonia in GBIF^9^ or plots of the Amazon Tree Diversity Network)^14^.

The updated list contains 1604 fewer species than our 2016 list and 3344 more than Cardoso *et al*.’s list. This reflects the removal from our 2016 checklist of 864 synonyms, misspellings or spelling variants, 1514 non-Amazonian species, and 301 non-trees (SI Appendix 3, species removed), a reduction of about 23% from our original list and corresponds to what we perceived as errors. A large portion of the “errors” still belong, according to our criteria, to an Amazonian tree species list, as discussed below. We also added 1075 tree species to the list, making the final list about 14% smaller than our original list. The new species include species listed by Cardoso *et al*. but not in our original list, newly published species, and tree species recorded in the Amazon Tree Diversity Network (ATDN) plots since 2016. Below we provide a more thorough account and discussion on these differences.

Finally, during the review we became aware of a number of new taxonomic treatments, which were taken into account in the updated list: *Crepidospermum* Hook.f. and *Tetragastris* Gaertn. were merged into *Protium* Burm.^15^; *Licania* Aubl. was split into eight genera^16^; *Buchenavia* Eichler was merged into *Terminalia* L.^17^; *Bribria* Wahlert & H. E. Ballard was taken out of *Rinorea* Aubl.^18^; and *Anaectocalyx* Triana*, Calycogonium* DC*, Catocoryne* Hook.f.*, Charianthus* D.Don*, Clidemia* D.Don*, Conostegia* D.Don*, Killipia* Gleason*, Leandra* Raddi*, Maieta* Aubl.*, Mecranium* Hook.f.*, Necramium* Britton*, Ossaea* DC*, Pachyanthus* A.Rich.*, Pleiochiton* Naudin ex A.Gray*, Sagraea* DC*, Tetrazygia* Rich. ex DC*, and Tococa* Aubl. were merged into *Miconia* Ruiz & Pav.^19^, and many *Psychotria* L. species have been transferred to *Palicourea* Aubl.^20,21^. The latter is still proceeding, so more changes are to be expected in the near future (P. Delprete pers. comm.).

### Synonyms

Eliminating synonyms is not a straightforward task, due to disagreements among literature sources. While our assessment supported Cardoso *et al*.’s hypothesis that synonyms inflated our 2016 checklist, it clearly showed that the inflation was not severe, and aroused partly from the use of taxonomic data aggregators, partly from genuine disagreement between taxonomic publications. Assuming that Cardoso *et al*.^4^ present the latest taxonomic consensus, and that taxonomic databases do not reflect that consensus, we have accepted most synonyms and spelling corrections they proposed. Although not in Cardoso *et al*., we also accepted the synonymy of *Virola calophylloidea* Markgr. into *Virola calophylla* (Spruce) Warb., based on the latest monograph^22^ of the genus and on Flora of Ecuador^23^. In total we removed 864 names from our list because of synonymy.

### Amazonian versus non-Amazonian species

Cardoso *et al*.^4^ hypothesized that 2757 tree species should be removed from our list because they do not occur in Amazonia. They based this on two arguments: (1) a different delimitation of the Amazonian forest, which is smaller than the one we used; (2) removal of tree species that are recorded in Amazonia according to the taxonomic literature sources used by the authors, but that have been collected more commonly in non-Amazonian biomes than in Amazonia.

We accepted Cardoso *et al*.’s^4^ first argument that the polygon used in^5^ comprises areas in the Andes and Guyana highlands. Consequently, we have refined our map of Amazonia to forested areas below 500 m elevation (SI Appendix, Fig. S1). We removed 1514 species present in the original list (13% of the total), either because their coordinates did not fall into our refined delimitation or because the records lacked coordinates and their presence in Amazonia could not be established beyond doubt.

From an ecological and empirical viewpoint, we do not agree with the second argument. Removing species whose median climatic values seem to be too cold or too dry to reflect presence in Amazonia – and consequently classifying them as non-Amazonian species – implies that individuals never disperse and establish outside the perceived niche/range of the species to which they belong. We acknowledge that most of the names removed by Cardoso *et al*. based on this argument are species whose primary populations are at higher elevations or in drier, more seasonal biomes adjacent to Amazonia (such as the Andes, the Llanos, the South American Open Diagonal, and the Atlantic Forest), but we keep the hypothesis of their occurrence in very low densities (as singletons in samples), in fact in line with all dynamical models since the Theory of Island Biogeography^24,25^. Furthermore this assumption of a climatic envelop eliminated erroneously several species of campinas and campinaranas, typical Amazonian vegetation types. Several of these taxa, namely 1172, are listed in FLORA (http://reflora.jbrj.gov.br/reflora/herbarioVirtual/), with Amazonian collections verified by the taxonomic specialist of their family. For example *Chamaecrista bahiae* (H.S.Irwin) H.S.Irwin & Barneby, which Cardoso *et al*. removed from the Amazonian list for being an Atlantic forest species^4^, has verified (det. Barneby) Amazonian collections in Herbário Virtual REFLORA (SI Appendix, Fig. S2) and *Virola carinata* (Spruce ex Benth.) Warb. has verified (det. W. Rodrigues) collections in Amazonian Igapó and Campinarana forest^22^.

### Trees versus non-trees

Among the 1,138 species in our check list pointed out by Cardoso *et al*. to represent non-tree growth forms, we found that ~300 (2.5% of the full list) were indeed never recorded as exhibiting tree growth. We removed these from the updated list. For the remaining species, the criticism is difficult to assess because there are no universal, ecologically justifiable distinctions between trees and shrubs^26^, and no authoritative databases of Amazonian plant habits. Cardoso *et al*. defined trees as “all species that reach ≥10 cm of diameter at breast height (dbh) during their life.” However, this is incompatible with the fact that growth form data in some taxa were checked against floras and checklists that do not use that definition. We retained ~850 of the species rejected by Cardoso *et al*. on the basis of growth form, either because they are classified as trees in Grandtner and Chevrette^27^ or because they have been confirmed to grow as trees ≥10 cm dbh, by field biologists and taxonomists, in the ATDN plots.

Amazonian tree inventories have documented a significant number of species that are typically observed as treelets, epiphytes, or lianas, occurring as free-standing trees ≥10 cm dbh (e.g., the shrub *Abuta grandifolia* (Mart.) Sandwith, the shrub-liana *Ampelozizyphus amazonicus* Ducke and the liana *Adenocalymma cladotrichum* (Sandwith) L.G.Lohmann). Since these ‘occasional’ trees participate in Amazonian tree communities, from an ecological point of view, we argue that they belong in a rigorous list of Amazonian trees.

### Taxonomically vetted

The validity of a species name, as well as all specimens assigned to that name, can be found in taxon revisions or monographs. In a taxonomically vetted checklist, this literature together with specialist opinion is the most important evidence for the occurrence of a species in a given region. Cases in which new material becomes available or specialists emit different opinion about either species validity or specimen determination raise different hypothesis about species occurrence. As an example among many, *Sloanea porphyrocarpa* Ducke (Type: Brasil. Pará, Obidos, 10 mar 1915, Ducke 20971), was considered a valid species in Amazonian Brazil and Bolivia by Palacios-Duque *et al*. in 2016^28^ but rejected by T. D. Pennington in that same year^29^. For this reason we also retained a number of species, considered synonyms by Cardoso *et al*. but considered “good” species by taxonomists in French Guiana (D. Sabatier. pers. obs). We added a column of verification to our list with three levels (and sublevels): 1 taxonomically verified presence (1a Cardoso *et al*.; 1b same but other name; 1c other); 2 Reported by Reflora (2a Amazonia; 2b Maranhão, Tocantins [presence in Amazonia less certain]); 3 not vetted (3a identified in ATDN plots, 3b collections in Amazonia in GBIF).

## Conclusion

The debate about the scale of Amazonian tree diversity is less the result of different levels of scholarly care than of different methodological choices of species inclusion criteria. The most important development is how these different hypotheses can inform future research. The central idea is that all species records should be annotated, including those not yet treated in taxonomic studies, for the sake of hypothesis testing.

The debate about which species merit inclusion in an Amazonian tree flora raises some important questions about the several thousand very rare species hypothesized to occur in Amazonian tree communities but not yet recorded there^7,30^. Are most of these taxa known species that will be discovered to occasionally spill-over into the margins of the Amazon basin from adjacent biomes? Or currently described species of shrubs or lianas that will be discovered to occasionally occur as trees? Alternatively, are they undescribed tree species that will eventually be discovered as locally endemic or extremely rare? These questions can be answered by monitoring changes in the Amazonian checklist over time, by increasing the vouchering of specimens during ecological surveys, data exchange and curation^31,32^, by digitization of specimens, support of taxonomic descriptions and monographs of families, in addition to field collecting that targets specific areas or taxa^5^.

We conclude that the list produced by Cardoso *et al*. should be considered extremely conservative in estimates of the species diversity of the region. The way forward towards a rigorous consensus list of Amazonian tree species is a system that reflects the different occurrence hypotheses by clearly showing the inclusion criteria underlying each species name in the list. By making the updated checklist publicly available, we hope to maintain a current list of Amazonian trees for both researchers and policymakers. Our list includes the latest taxonomy updated from^4^, with notes which specialist in their team assessed the name, and both the estimated total number of trees in Amazonia for those trees species occurring in the ATDN plots and the number of collections we found in our survey. Based on this users can make a judgement about the validity of the name, and commonness and rarity of tree species and the likelihood of encountering such a species.

## Methods

We examined the five potential sources of error in our tree species list cited by Cardoso *et al*.: “(i) 1,514 demonstrably non-Amazonian species; (ii) individual species listed more than once, as synonyms and spelling variants (864 names); (iii) listing of non-tree species, including herbs, shrubs, vines, and epiphytes (301 names); (iv) the inclusion of Old World species not native to the Neotropics (96 names); and (v) the inclusion of non-Amazonian cultivated species (53 names).”

The inclusion of species not native to the Neotropics, cultivated species, and misspelled species—reflect clear errors made during the construction of the original list. To correct those errors, we removed all of those species from the updated list.

Other sources of error cited by Cardoso *et al*.—species in our list that they considered to be non-Amazonian, non-trees, or synonyms—are harder to address, since they reflect differences in opinion over what constitutes an Amazonian tree, or a taxonomically accepted name. In these cases, we examined the validity of Cardoso *et al*.’s opinions as follows. To assess whether non-Amazonian species fide Cardoso *et al*. deserved removal from the checklist, we compared our Amazonian delimitation with theirs and reviewed the statistical analysis they used to define ‘non-Amazonian.’ To assess whether non-tree species fide Cardoso *et al*. have never been recorded as trees, we first evaluated whether any of these had been confirmed as trees in the ATDN plots in lowland Amazonia, and then used the growth forms of Grandtner and Chevrette^27^ to settle the remainder.

Finally, we added new tree species not present in our 2016 list but listed in Cardoso *et al*., e.g.^33^, new tree species added in the ATDN plot database, and newly published tree species, such as^34,35^. Finally, to assess whether names considered to be synonyms by Cardoso *et al*. were widely accepted as synonyms, we checked them against Tropicos, the online database used to generate the 2018 checklist of all vascular plants in the Americas^36^.

## Supplementary information

Supplementary information to the manuscript “Towards a dynamic list of Amazonian tree species”

Appendix 1
