# Supplementary material for: Towards a dynamic list of Amazonian tree species
Source: Sci Rep. 2019 Mar 5;9:3501. doi: 10.1038/s41598-019-40101-y (PMC6401171; doi:10.1038/s41598-019-40101-y)
Supplement: Supplementary file 1 — Supplementary information to the manuscript “Towards a dynamic list of Amazonian tree species” [file 41598_2019_40101_MOESM1_ESM.pdf]

Supplementary information to the manuscript "Towards a dynamic list of Amazonian tree species"

Hans ter Steege[1,2,3*], Sylvia Mota de Oliveira[1], Nigel C. A. Pitman[4], Daniel Sabatier[5], Alexandre Antonelli[6,7,8,9], Juan E. Guevara Andino[4,10,11] , Gerardo A. Aymard[12] & Rafael P. Salomão[2,13]

[1]Naturalis Biodiversity Center, Vondellaan 55, Postbus 9517, 2300 RA Leiden, The Netherlands.

[2]Coordenação de Botânica, Museu Paraense Emílio Goeldi, Av. Magalhães Barata 376, C.P. 399, Belém, PA 66040–170, Brazil.

[3]Systems Ecology, Free University Amsterdam, The Netherlands.

[4]Science and Education, The Field Museum, 1400 S. Lake Shore Drive, Chicago, IL 60605–2496, USA.

[5]AMAP, IRD, CIRAD, CNRS, Université de Montpellier, INRA, Boulevard de la Lironde, TA A-51/PS2, F-34398 Montpellier Cedex, France.

[6]Department of Biological and Environmental Sciences, University of Gothenburg, Box 461, SE-405 30 Göteborg, Sweden.

[7]Gothenburg Botanical Garden, Carl Skottsbergsgata 22A, SE-413 19, Göteborg, Sweden.

[8]Gothenburg Global Biodiversity Centre, Box 461, SE-405 30 Göteborg, Sweden.

[9]Department of Organismic and Evolutionary Biology, Harvard University, 26 Oxford St., Cambridge, MA 02138 USA.

[10]Yachay Tech, School of Biological Sciences, Hacienda San José s/n, San Miguel de Urcuquí, Ecuador.

[11] Grupo de Investigación en Biodiversidad, Medio Ambiente y Salud-BIOMAS- Universidad de las Américas, Campus Queri, Quito Ecuador

[12]UNELLEZ-Guanare, Programa de Ciencias del Agro y el Mar, Herbario Universitario (PORT), Mesa de Cavacas, Estado Portuguesa 3350, Venezuela; current address: Compensation International Progress S.A., P.O. Box 260161, Bogotá, D. C., Colombia

[13]Programa Professor Visitante Nacional Sênior na Amazônia – CAPES. Universidade Federal Rural da Amazônia, Av. Perimetral, s/n, Belém, PA, Brazil.

Correspondence and requests for materials should be addressed to H.T.S. (email: hans.tersteege@naturalis.nl)

Contents

1. Figure S1

2. Figure S2

3. Legend to appendix 1.

4. References

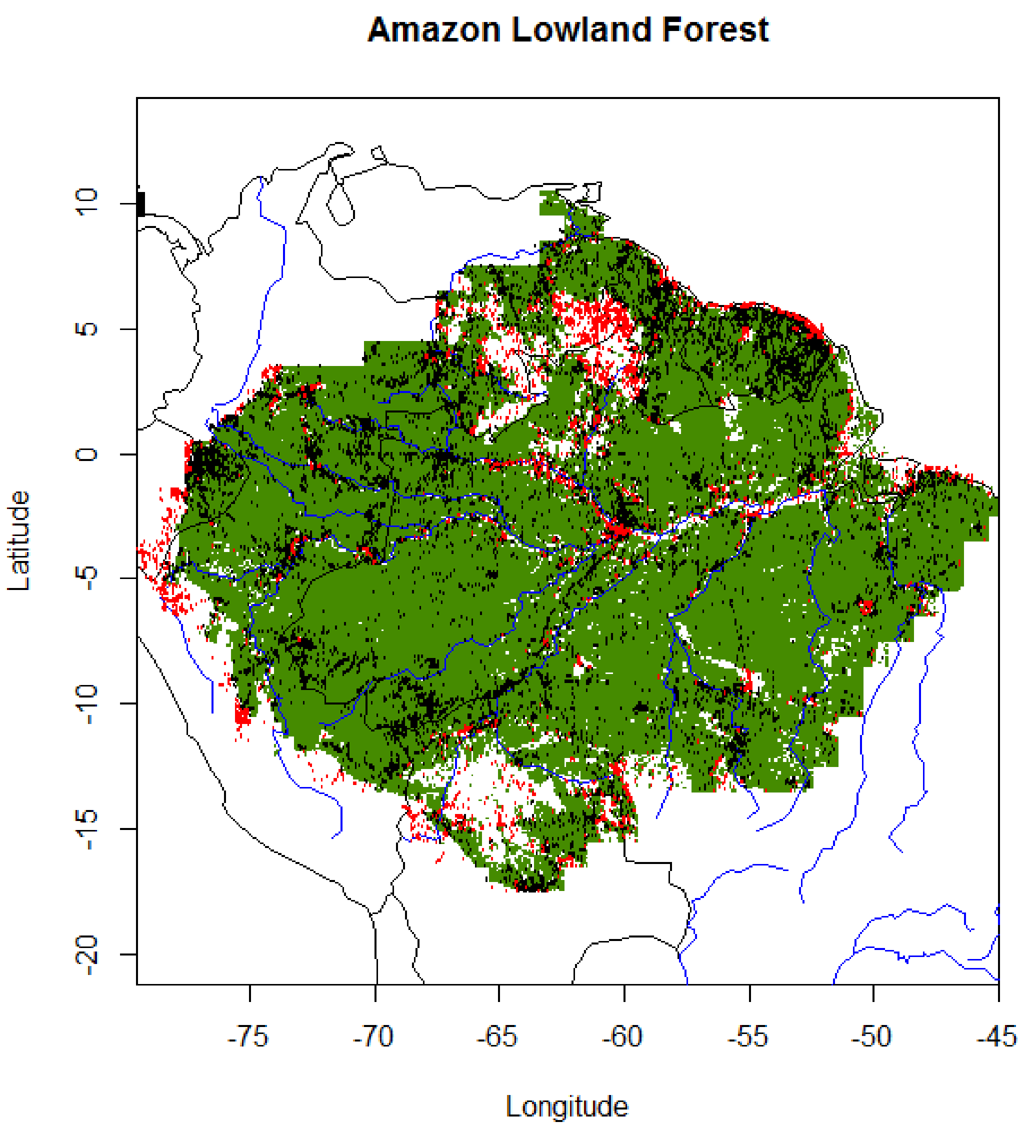

Fig. S1. Map of Amazonian Lowland Forest. The green area represents land area [1] minus large water bodies [1], areas over 500 m elevation [2], and areas originally without forest [3,4]. Black dots: collections used in [5] for a checklist of Amazonian trees. Red dots: collections removed based on location outside Amazon lowland forest area. Map created with custom R[6] script. Base map source (country.shp, rivers.shp): ESRI (http://www.esri.com/data/basemaps, © Esri, DeLorme Publishing Company).

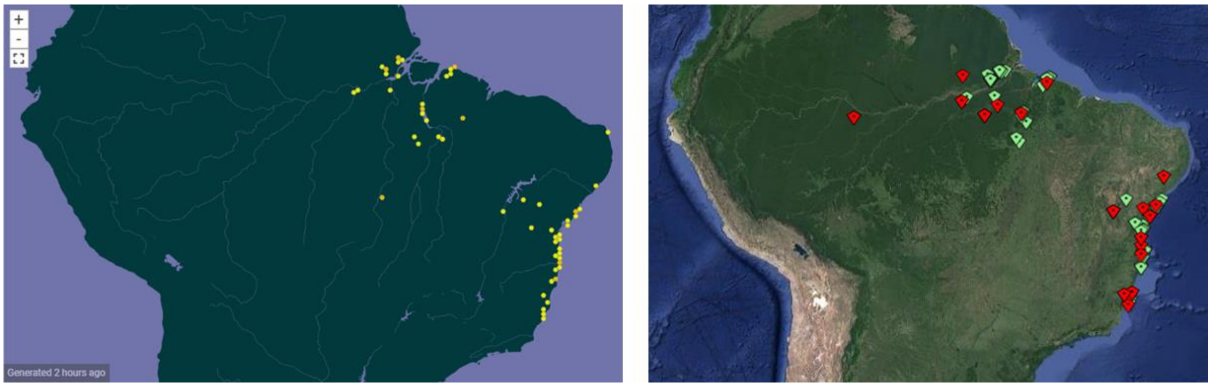

Fig. S2. Collections of *Chamaecrista bahiae* (H.S.Irwin) H.S.Irwin & Barneby. **Left**: All collections of *Chamaecrista bahiae* extracted from GBIF (Chamaecrista bahiae (H.S.Irwin) H.S.Irwin & Barneby in GBIF Secretariat (2017). GBIF Backbone Taxonomy. Checklist dataset https://doi.org/10.15468/39omei accessed via GBIF.org on 2018-07-14.). **Right**: All collections of *Chamaecrista bahiae* extracted from Herbário Virtual REFLORA (http://reflora.jbrj.gov.br/reflora/herbarioVirtual/, accessed February 5, 2018 (CC-BY SA 4.0: https://creativecommons.org/licenses/by-sa/4.0/); map data: Google, ©2018 NASA terrametrics).

3. Legend Appendix Checklist Amazon trees 2018

**Checklist 2018**	Species name in 2018 checklist (bold also in Cardoso et al 2017 [7])
**Author**	Author of Species name in 2018 checklist
**Taxonomic verification**	1 taxonomically verified presence (1a Cardoso et al; 1b same but other name; 1c other). 2a Reported for Amazonia in Reflora; 2b reported Maranhão, Tocantins in Reflora. 3a identified in ATDN plots, 3b collections in Amazonia in GBIF
**new name**	Is the name new with respect to the list of ter Steege et al 2016 [5]?
**Revised by**	revised by … in Cardoso et al 2017, and others
**tree.10cm.DBH Cardoso**	Is the species considered a tree by Cardoso et al 2017? (1 = yes)
**non.Amazonian Cardoso et al**	Is the species considered non-Amazonian by Cardoso et al 2017 (1 = yes)
**note**	Additional notes
**collections in Amazon**	Number of GBIF collections (modified from ter Steege et al 2016) in lowland Amazonian forest area (see Fig. S1)
**nr. ind. in ATDN plots**	Nr of tree individuals in ATDN plots in 2018 (courtesy ATDN 2018) [8,9]
**est. population in Amazon**	Population size in Amazon (^{10}log classes: 4 = 10,000-99,000 …. 9 = 1^9 - 9.9^9) (courtesy ATDN 2018) [9]

Sheet 2 and 3 list the synonyms changes that were applied to the ter Steege et al 2016 list (with sources for the synonymy change), and the species that were deleted from that list, respectively.

4. References

1 Environmental Systems Research Institute. ESRI Data & Maps 1999. An ESRI White Paper. (Environmental Systems Research Institute, Redlands, US, 1999).

2 Jet Propulsion Laboratory. *Shuttle Radar Topography Mission*, 2009).

3 Soares-Filho, B. S. *et al.* Modelling conservation in the Amazon basin. *Nature* **440**, 520-523, doi:http://www.nature.com/nature/journal/v440/n7083/suppinfo/nature04389_S1.html (2006).

4 Soares-Filho, B. S. *et al.* (ed Oak Ridge Oak Ridge National Laboratory Distributed Active Archive Center, Tennessee, U.S.A.) (http://daac.ornl.gov//LBA/guides/LC14_Amazon_Scenarios.html, 2013).

5 ter Steege, H. *et al.* The discovery of the Amazonian tree flora with an updated checklist of all known tree taxa. *Scientific Reports* **6**, 29549, doi:10.1038/srep29549 (2016).

6 R: A language and environment for statistical computing (R Foundation for Statistical Computing, Vienna, Austria, 2017).

7 Cardoso, D. *et al.* Amazon plant diversity revealed by a taxonomically verified species list. *Proceedings of the National Academy of Sciences* **114**, 10695-10700, doi:10.1073/pnas.1706756114 (2017).

8 ter Steege, H. *et al.* Hyperdominance in the Amazonian Tree Flora. *Science* **342**, 1243092, doi:10.1126/science.1243092 (2013).

9 Amazon Tree Diversity Network. <http://web.science.uu.nl/Amazon/ATDN/> (2018).
